# Germans learn how to save lives: a nationwide CPR education initiative

**DOI:** 10.1186/s12245-018-0171-1

**Published:** 2018-02-17

**Authors:** Manuela Malsy, Richard Leberle, Bernhard Graf

**Affiliations:** Department of Anesthesiology, University Medical Center Regensburg, Franz Josef Strauss Allee 11, 93053 Regensburg, Germany

**Keywords:** Resuscitation, Sudden cardiac arrest, Lay resuscitation, Resuscitation Week, Kids Save Lives

## Abstract

**Background:**

Sudden cardiac death is one of the most frequent causes of death in Germany and the third leading cause of death in the industrialized world. Yet, the percentage of people providing first aid in the case of sudden cardiac arrest in Germany is alarmingly low by international comparison. Training Germans or reminding them of the simple but effective steps of resuscitation, so that everybody can save a live in an emergency.

**Methods:**

For the campaign ‘Resuscitation Week’, physicians and paramedics trained passers-by in cardiovascular resuscitation free of charge. Skills were evaluated before and after the instruction by means of a questionnaire.

**Results:**

Three hundred three people aged between 9 and 89 years were trained and evaluated. Forty-nine passers-by had never participated in a resuscitation course, and 46.8% had participated in a course more than 20 years ago. Before the instruction, 41.6% of the passers-by were confident to be capable of resuscitating a person; after the instruction, however, this percentage had risen to 100%!

**Conclusions:**

Saving a life is simple, but one has to know what to do in the case of sudden cardiac arrest. The German population is being gradually trained in resuscitation using campaigns such as ‘Resuscitation Week’ and ‘Kids Save Lives’ to break down barriers in the long term. However, lives are not only saved by training but also by refreshing knowledge and skills; thus, a further effective approach may be training all holders of a driving license in cardiopulmonary resuscitation in intervals of 5 years.

## Background

Sudden cardiac death is one of the most frequent causes of death in Germany and the third leading cause of death in the industrialized world [1]. In Germany alone, about 70,000 to 80,000 people are affected by this disease, which constitutes about 1% of the population [2]. However, the percentage of people providing first aid in the case of sudden cardiac arrest in Germany is alarmingly low. For this reason, only about 45% of affected patients arrive at the hospital alive, where many of them die despite intensive therapeutic intervention [3].

Sudden cardiac death is caused by malign arrhythmia due to coronary heart disease or dilated cardiomyopathy [4]. Less frequent causes are myocarditis, hypertrophic cardiomyopathy, right ventricular cardiomyopathy, and channelopathies such as the Brugada syndrome, the long QT syndrome, and the short QT syndrome [5]. Patients lose consciousness and die within 1 h after the onset of acute symptoms [6]. Because the average ambulance response time in Germany is about 8 to 10 min, telephone-assisted resuscitation (T-CPR) has been introduced in accordance with the 2010 guidelines on cardiopulmonary resuscitation [7]. In the case of suspected cardiac arrest, the caller is assisted in the resuscitation process by the dispatcher of the emergency service via the phone [8]. Since the introduction of T-CPR, the rate of first aid provided by lay people witnessing sudden cardiac arrest has significantly increased [9]. Fear of doing something wrong or other inhibitions still prevent many people from starting chest compression. In the past years, the rate of cardiopulmonary resuscitation performed by laypeople in Germany was significantly lower than in other countries [10]. In the Netherlands and Scandinavia, for instance, partly more than 60% of chest compressions in the case of sudden cardiac arrest were performed by first responders before the arrival of the ambulance services [10, 11]. In these countries, the population is regularly trained in resuscitation procedures [12]. Comprehensive nationwide training of the population may thus also permanently increase the number of cardiopulmonary resuscitation performed by laypeople in Germany, thus significantly improving the survival probability of the affected patients as well as their quality of life [1].

The objective of the project was to train Germans or remind them of the simple but effective steps of cardiopulmonary resuscitation free of charge, so that everybody can save a live in an emergency situation.

## Methods

Physicians and paramedics trained interested people free of charge in cardiovascular resuscitation according to the motto ‘We learn how to save lives’. The emphasis is on the recognition of a cardiac arrest, the activation of help, and alerting the emergency service as well as the understanding and effective implementation of the chest compression. The latter involves finding the correct pressure point, the correct frequency, and the right depth of compression. Skills were assessed by means of a questionnaire before and after the instruction.

Questionnaire 1 (before the instruction)

- How old are you?
- Are you a man or a woman?
- Have you ever taken part in a cardiopulmonary resuscitation course (for instance when taking your driver’s license, as an in-house first aider in your company, in the context of voluntary work, or others)?
- What year did you last participate in a cardiopulmonary resuscitation course?
- Do you believe yourself capable of performing chest compression in an emergency?

Questionnaire 2 (after the instruction)

- Do you believe yourself capable of performing chest compression in an emergency?

## Results

**Fig. 1:**
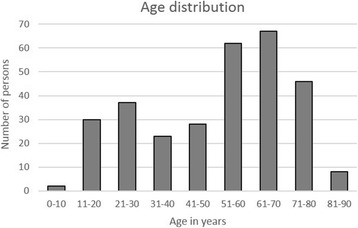
Age distribution of evaluated persons

**Fig. 2:**
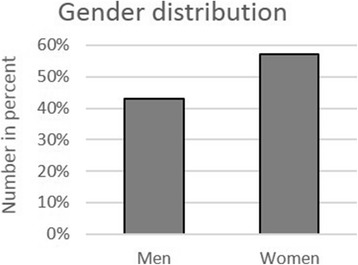
Gender distribution by percentage

**Fig. 3:**
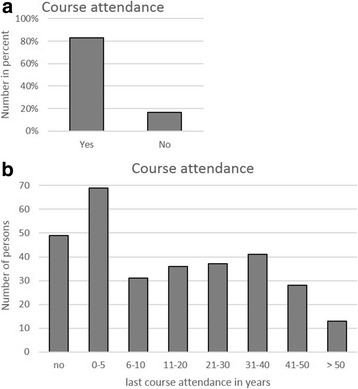
Course attendance (**a**) and last course attendance in years (**b**)

**Fig. 4:**
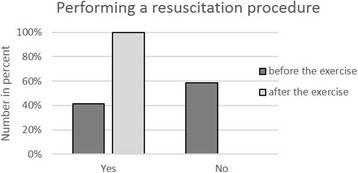
Performing a resuscitation procedure

Overall, 303 persons were trained and assessed. The age of the participants ranged between 9 and 89 years (Fig. 1); 42.9% were men and 57.1% women (Fig. 2). 16.2% of the passers-by had never participated in a cardiopulmonary resuscitation course (Fig. 3a), 46.8% had attended a course more than 20 years ago, and 32.3% more than 30 years ago (Fig. 3b). Before the instruction, 41.6% of the passers-by were confident to be capable of resuscitating a person; after the instruction, however, this percentage had risen to 100% (Fig. 4)!

## Discussion

Oxygen deprivation of the brain causes neurological damage already 4 to 5 min after sudden cardiac arrest [13]. However, the average ambulance response time in Germany is 8 to 10 min. This critical time frame may only be overcome by the support of first responders starting cardiovascular resuscitation [14]. This was precisely the point at which the German Federal Ministry of Health addressed the problem by initiating the campaign ‘Resuscitation Week’ based on the motto ‘Save A Life. 100 Per Resuscitation’ [15].

Passers-by are trained to recognize a cardiac arrest, to activate help and to alert the emergency service, and to perform the chest compression effectively. Furthermore, the targets of this campaign are also to raise the awareness of every single person to perform life-saving procedures, breaking down inhibitions, and informing on life-saving emergency measures. Only regular hands-on practice enables people to competently perform live-saving procedures in emergency situations [16]. 41.6% of the assessed passers-by believed themselves capable of resuscitating a person, and this rate is significantly higher than the actually measured percentage of first responders [10]. The reason for this discrepancy may be that participation in the refresher course was voluntary and that only really interested passers-by participated in the instruction that was also free of charge.

Nearly every participant considered helping in an emergency situation natural; yet, 177 of 303 passers-by had never participated in a resuscitation course before or no longer considered themselves capable of performing chest compression. 83.2% of the population has been trained in resuscitation, either when taking their driving license, in the context of voluntary work, or as an in-house first aider in their company. This percentage reflects the good quality of basic First Aid-training in the general population in Germany. The real problem is refresher courses because 46.5% of the passers-by had attended their last resuscitation course more than 20 years ago. 33% of the passers-by had participated in a course in the past 10 years; thus, this group of people can be considered capable of performing cardiovascular resuscitation in emergency situations. The campaign ‘Resuscitation Week’-initiated by the German Association of Anesthesiologists and the German Society of Anesthesiology and Intensive Care Medicine in cooperation with the German Resuscitation Council and the non-profit foundation German Anesthesiology in 2013-seeks to raise the awareness of resuscitation and to refresh already existing skills [15].

Additionally, based on the resolution of the Conference of German cultural ministers in 2014, two lessons on resuscitation have been implemented in the curriculum of all secondary schools from year 7 onwards [17]. The initiative ‘Kids Save Lives’ aims at improving the resuscitation rate of laypeople in Germany by increased training. Next to the direct advantage of instructing pupils in basic cardiopulmonary resuscitation, pupils trained in resuscitation may act as multipliers in their own families and thus reach further parts of the population [18]. The training of pupils in resuscitation is now part of the school curriculum. The implementation of the training is based, among others, on the curriculum of the German Resuscitation Council and the Federal Association for First Aid of aid organizations [19, 20]. The emphasis is on the recognition of a cardiac arrest, the activation of help, and alerting the emergency service as well as the understanding and effective implementation of the chest compression. The instruction of teachers of biology and sports is carried out by medical staff.

## Conclusions

Saving a life is simple, but one has to know what to do if a person suffers sudden cardiac arrest. The German population is being gradually trained in resuscitation by means of campaigns such as ‘Resuscitation Week’ and ‘Kids Save Lives’ to break down barriers in the long-term and to refresh already existing skills. The objective of this campaign is to train people in the simple but effective steps of resuscitation, so that everybody can save a live in an emergency. This way, more people may survive and enjoy better quality of life after resuscitation. However, life-saving skills need to be refreshed at regular intervals; thus, a further effective approach may be training all holders of a driving license in cardiopulmonary resuscitation in intervals of 5 years.

## References

[1] Wnent J, Geldner G, Werner C, Bottiger BW, Fischer M, Scholz J, Grasner JT (2014). Bad boller resuscitation talks: 10 basic ideas for 10,000 lives. Anasthesiol Intensivmed Notfallmed Schmerzther.

[2] Fischer M, Lang S, Wnent J, Seewald S, Brenner S, Jantzen T, Bohn A, Gräsner JT (2017). Das reanimationsfreie Intervall bestimmt das Kurz- und Langzeitüberleben – eine Analyse aus dem Deutschen Reanimationsregister. Anästh Intensivmed.

[3] Estner HL, Günzel C, Ndrepepa G, William F, Blaumeiser D, Rupprecht B, Hessling G, Deisenhofer I, Weber MA, Wilhelm K, Schmitt C, Schömig A (2007). Outcome after out-of-hospital cardiac arrest in a physician-staffed emergency medical system according to the Utstein style. Am Heart J.

[4] Hayashi M, Shimizu W, Albert CM (2015). The spectrum of epidemiology underlying sudden cardiac death. Circ Res.

[5] Zipes DP, Wellens HJ (1998). Sudden cardiac death. Circulation.

[6] Vandenberg JI, Perry MD, Hill AP (2017). Recent advances in understanding and prevention of sudden cardiac death. F1000Res.

[7] Nolan JP, Soar J, Zideman DA, Biarent D, Bossaert LL, Deakin C, Koster RW, Wyllie J, Böttiger B, ERC Guidelines Writing Group (2010). European resuscitation council guidelines for resuscitation 2010 section 1. Executive summary. Resuscitation.

[8] Dameff C, Vadeboncoeurb T, Tully J, Panczykc M, Dunhama A, Murphya R, Stolze U, Chikania V, Spaitee D, Bobrowa B (2014). A standardized template for measuring and reporting telephone pre-arrival cardiopulmonary resuscitation instructions. Resuscitation.

[9] Bohm K, Vaillancourt C, Charette ML, Dunford J, Castrén M (2011). In patients with out-of-hospital cardiac arrest, does the provision of dispatch cardiopulmonary resuscitation instructions as opposed to no instructions improve outcome: a systematic review of the literature. Resuscitation.

[10] Wnent J, Bohn A, Seewald S, Fischer M, Messelken M, Jantzen T, Gräsner I, Gräsner JT (2013). Bystander resuscitation: the impact of first aid on survival. Anasthesiol Intensivmed Notfallmed Schmerzther.

[11] Strömsöe A, Svensson L, Claesson A, Lindkvist J, Lundström A, Herlitz J (2011). Association between population density and reported incidence, characteristics and outcome after out-of-hospital cardiac arrest in Sweden. Resuscitation.

[12] Wissenberg M, Lippert FK, Folke F, Weeke P, Hansen CM, Christensen EF, Jans H, Hansen PA, Lang-Jensen T, Olesen JB, Lindhardsen J, Fosbol EL, Nielsen SL, Gislason GH, Kober L, Torp-Pedersen C (2013). Association of national initiatives to improve cardiac arrest management with rates of bystander intervention and patient survival after out-of-hospital cardiac arrest. JAMA.

[13] Perkins GD, Handley AJ, Koster RW, Castrén M, Smyth MA, Olasveengen T, Monsieurs KG, Raffay V, Gräsner JT, Wenzel V, Ristagno G, Soar J (2015). European Resuscitation Council Guidelines for Resuscitation 2015: Section 2. Adult basic life support and automated external defibrillation. Resuscitation.

[14] Schroeder DC, Ecker H, Wingen S, Semeraro F, Böttiger BW (2017). “Kids Save Lives”—resuscitation training for schoolchildren: systematic review. Anaesthesist.

[15] Van Aken H, Böttinger BW, Schleppers A (2013). Woche der Wiederbelebung Ein Leben Retten. 100 Pro Reanimation. Notf Med Up2date.

[16] Abolfotouh MA, Alnasser MA, Berhanu AN, Al-Turaif DA, Alfayez AI (2017). Impact of basic life-support training on the attitudes of health-care workers toward cardiopulmonary resuscitation and defibrillation. BMC Health Serv Res.

[17] Beschluss 395. Schulausschluss der Kultusministerkonferenz 2014.

[18] Böttiger B, Nöldge-Schomburg G, Rücker G, Van Ackern K, Van Aken H (2014). Wiederbelebung als Pflichtthema in den Schulen, Berufsverband Deutscher Anästhesisten (BDA).

[19] Recommended curriculum to teach and train resuscitation to school children in Germany.

[20] Bohn A, Van Aken H, Mollhoff K (2012). Teaching resuscitation in schools: annual tuition by trained teachers is effective starting at age 10. A four-year prospective cohort study. Resuscitation.

